# Physical activity and associated factors among pregnant women in Ethiopia: facility-based cross-sectional study

**DOI:** 10.1186/s12884-020-2777-6

**Published:** 2020-02-10

**Authors:** Teklehaimanot Tekle Hailemariam, Yosef Sibhatu Gebregiorgis, Berihu Fisseha Gebremeskel, Tsiwaye Gebreyesus Haile, Theresa Monaco Spitznagle

**Affiliations:** 1 Department of Physiotherapy, School of Medicine, College of health sciences, and Ayder comprehensive specialized hospital, Mekelle University, P.O. Box - 1871, Tigrai, Mekelle, Ethiopia; 2 Department of Epidemiology, School of Public health, College of health sciences, Mekelle University, P.O. Box - 1871, Tigrai, Mekelle, Ethiopia; 3 School of Medicine, Program in Physical Therapy, Washington University, St Louis, MO USA

**Keywords:** Physical activity, Maternal health, Pregnancy, Exercise, Mekelle

## Abstract

**Background:**

Regular physical activity (PA) has health benefits, including reducing the risk of complications during pregnancy. In Ethiopia, little is known about PA status and its determinants among pregnant women. The purpose of this study was to assess PA status and associated factors among pregnant women attending antenatal care at public and private health facilities in Mekelle, Ethiopia.

**Methods:**

A facility-based cross-sectional study was conducted. Data was collected from 299 pregnant women using a structured questionnaire. Study participants were selected using a simple random sampling technique. A binary logistic regression was modeled to investigate the statistical significance of independent variables with PA status during pregnancy. Factors associated with PA status were estimated using adjusted odds ratios with 95% confidence intervals and statistical significance was declared at *p*-value < 0.05.

**Results:**

79.3% of the study participants were classified as sedentary. The age group of 26–35 years (AOR: 2.69, 95% CI: 1.07–6.78), attending non-formal education (AOR: 13.50, 95% CI: 2.65–68.91), and women who did not work outside the home (AOR: 5.23, 95% CI: 1.34–20.38) were significantly associated with a higher risk of sedentary activity status. Pregnant women who were married (AOR: 0.26, 95% CI: 0.09–0.73), had two children (AOR: 0.13, 95% CI: 0.03–0.59), traveled an hour or more to health facilities (AOR: 0.31, 95% CI: 0.11–0.89) were protected from being sedentary.

**Conclusion:**

Sedentary PA status was highly prevalent during pregnancy. Pregnant women in the age group of 26–35 years, with a non-formal education, and women who did not work outside the home had a greater risk of reporting being sedentary. Those who were married, had two children, and traveled an hour or more to health facilities were less likely to be sedentary. Stakeholders (Tigrai regional health bureau, Mekelle University, local NGOs working with pregnant women and societies at large) should give higher emphasis on designing appropriate strategies including educational interventions to overcome barriers to PA during pregnancy.

## Background

Physical activity (PA) is defined as any bodily movement produced by skeletal muscles that require energy expenditure [1]. According to the American College of Obstetrics and Gynecology (ACOG), pregnant women achieve at least 150 min or more per week of moderate-intensity PA they are labeled as physically active, otherwise, they are classified as sedentary [2]. Reduced PA is rising across many countries in the world with major health implications including increased non-communicable diseases (NCDs) such as cardiovascular disease, diabetes and cancer and their shared common risk factors like raised blood pressure, raised blood sugar and overweight, and it has been identified as a risk factor for 6% of all deaths, worldwide [3]. In general, a quarter of the world’s population in particular women compared to men do not meet the minimal PA recommendations that signifying physically inactive [4].

Pregnancy is a period in women’s lives characterized by intense physiological, physical and psychological changes in which maternal systems adapt to accommodate the increasing demands of fetal growth and development [5, 6]. These adaptational changes can lead to various pregnancy-induced health problems such as low back pain, gestational diabetes mellitus (GDM), hypertension, pre-eclampsia, fetal growth restriction, urinary incontinence, mental disorders, and maternal obesity [6, 7]. Pregnant women who engage in the prescribed PA requirements during their pregnancies reduced the risk of the aforementioned health problems [3, 6–10]. Several studies have also documented other health benefits of increased PA during pregnancy, include decreasing the incidence of preterm birth [5] and cesarean deliveries [8], improved cardiovascular function [9], improvement or maintenance of physical fitness, reduced symptoms of depression [10], and enhanced psychologic well-being [2]. Nevertheless, many women tend to decrease instead of maintain or increase their PA during pregnancy [11–22].

The rates of physical inactivity (women who did not achieve the minimum PA recommendation) during pregnancy range between 64.5 and 91.5%, and tend to be higher in the third trimester of pregnancy [5–8, 23].

Reported factors associated with a higher risk for inactivity or decreased PA during pregnancy across the globe are varied, including older age, low-income, low level of education, poor health status, [15, 24, 25], fear of miscarriage [17], inadequate knowledge [1, 15, 26], lack of supportive environment, [27], physical changes [1, 15], and increased parity [25].

Traditionally it has been thought that, in African and other low-income regions, there is a high physical workload associated with household activities and thus pregnant women who live in low- income regions are more physically active [28]. However, recent studies in Nigeria, Zambia, and South Africa have demonstrated the opposite perspective, women are now reporting reduced PA during pregnancy in these regions [25, 29, 30]. In addition, in each of these regions in Africa, similar factors associated with reduced PA have been reported during pregnancy. Older age, lower education and income, history of smoking, poor health status, and excess weight gain among African women has been found to be associated with perceived decrease participation in PA [1, 14, 15, 17, 19, 25–27].

In Ethiopia, little is known concerning the PA status and associated factors (maternal weight control and fitness, alleviating pregnancy-related pain and psychological symptoms) among pregnant women. A single study conducted in Jimma Ethiopia by Hjorth et al. [31] revealed that 76.4% of pregnant women surveyed reported spending most of their time doing sedentary activities, including eating, sitting, sleeping/resting in bed and cooking. However, no other research has been executed on this topic in Ethiopia. Thus, the purpose of this study was to assess PA status and associated factors among pregnant women attending antenatal care at public and private health facilities in Mekelle, Ethiopia.

## Methods

### Study design and setting

A facility-based cross-sectional study was conducted from January 10, 2018, to February 10, 2018, in Mekelle, Ethiopia. Mekelle is a city located around 780 km north of the Ethiopian capital, Addis Ababa, at an elevation of 2084 m above sea level, in the region of Tigrai. The Tigrai regional state has five zones. Mekelle city being designated as the regional capital city. Mekelle is administratively divided into seven sub-cities in which the entire Tigrai population obtains their specific healthcare services. Mekelle has three public hospitals, nine health centers, four private hospitals, more than 30 private clinics, and one ortho-physiotherapy center. Of these, 12 public and 7 private health facilities were providing antenatal care services.

Mekelle has a total population of 358,529 of whom 176,986 are females [32]. The Ethiopian Demographic and Health Survey (EDHS 2016) reported a countrywide fertility rate of 4.6, 42, and 33% employment status for women. However, in Tigrai, this national report revealed a similar fertility rate 4.7% and a slightly higher, literacy rate 51%, and employment status 37.4% of women. In addition, in Tigrai, health facility delivery rate is 56.9% (716 births per 1129 interviewed women) with the same proportion (56.5%) gave births in public health facilities followed by at home (41.0%). Furthermore, 59.3% of births were delivered by a skilled provider, and 49.6% of these births were attended by nurse or midwife followed by traditional birth attendants (23.2%) [33].

### Study population

Pregnant women during any trimester of pregnancy who were attending antenatal care during the data collection period at randomly selected public (12) and private health facilities (7).

### Inclusion criteria

Study participants who were having antenatal care during the data collection period in the selected public and private health facilities.

### Exclusion criteria

Pregnant women who presented with medical or obstetric complications and serious psychological conditions that could have an impact on the reliability of data/information were excluded.

### Sample size, sampling techniques, and procedures

The required sample size was determined using a single population proportion formula based on the assumption of a 95% confidence interval with a margin of error of 5%. A simple random sampling technique was used to select the eight public and private health facilities out of 19 utilized for data collection. These health facilities were chosen because they provide antenatal services and report to the Tigrai Regional Health Bureau (TRHB). According to the Federal Democratic Republic of Ethiopia Ministry of Health; health and health-related indicators in 2016/2017 report indicated that in Tigrai, 125,373 (69.5%) of women had at least four antenatal visits during their last pregnancy, and 118,219 (65.5%) of births was delivered by skilled attendants [34].

**Fig. 1:**
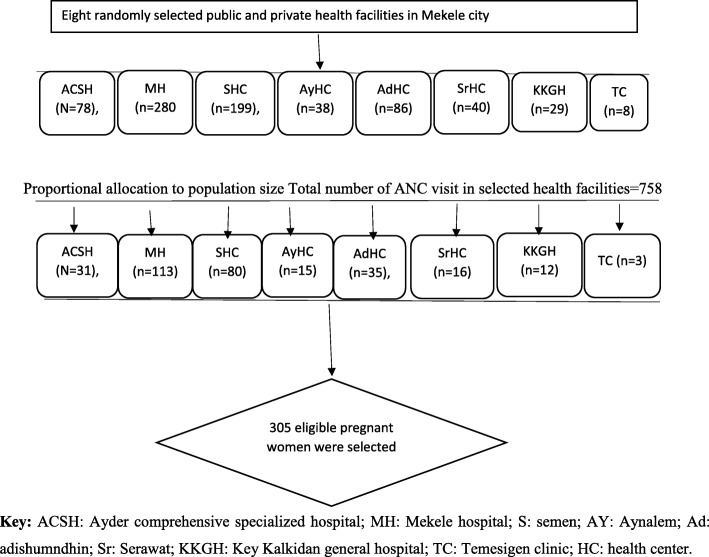
Schematic presentation of sampling procedures of Physical activity and associated factors among pregnant women in Ethiopia

The number recruited from each health facility was determined based on a population-based proportion that was developed considering the TRHB 2009 Ethiopian Fiscal Year (EFY) annual report and recent data reported by the health institutions for the third quarter of the previous year. All eligible pregnant women attending their antenatal care visits at selected health facilities during the study period were randomly approached for inclusion until the total sample size was attained. A total of 305 participants was targeted based on these calculations (Fig. 1).

### Data collection instrument and methods

Data were collected using a structured questionnaire developed from reviewing relevant literature. The questionnaire contains socio-demographic, obstetric and health, physical activity characteristics, and Pregnancy Physical Activity Questionnaire (PPAQ) which was developed by Chasan-Taber et al. [35]. A slight modification was made on PPAQ tool; for instance, two items like “playing with pets, and “mowing the lawn by riding a mower”, and by using a walking mower, raking, and gardening” were omitted in the present study’s tool due to cultural and feasibility issues in the Ethiopian context. Four experts (one obstetric and gynecologist, two chief physiotherapy specialists and one chief midwifery) with good knowledge of the subject matter of the study’s theme and both language versions had contextually constructed the contents of the original and local language versions of the PPAQ.

A questionnaire was translated into Tigrigna which is entirely an official language spoken in the Tigrai region, Ethiopia. This questionnaire was pretested on 5% (15) of pregnant women who were not part of the study participants from similar health settings and based on pretesting feedback modifications were made to the survey prior to administration.

For each activity, respondents were asked to select the category that best approximates the amount of time spent on that activity per day or week during the current trimester. At the time of recruitment, the women were informed about the purposes and procedures of this study. After highlighting the introduction and receiving oral and written consent, the participants completed the questionnaires for over 15 min in a quiet place in the clinic.

The PPAQ measures the frequency and duration of activities and gives an intensity value to each activity developed by Chasan-Taber et al. (31). The calculation was total activity = sum of (duration * intensity) for each question. Each activity was classified according to intensity in Metabolic Equivalent Task (MET)—sedentary (< 1.5 MET), low (1.5 to < 3.0 MET), moderate (3.0 to 6.0 MET), and vigorous-intensity (> 6.0 MET)—and type—labor, domestic (e.g., caring for a person), and sports/exercise [35].

### Data processing and analysis

After the collection of data, all collected questionnaires were checked for completeness, correctness, and internal consistency to exclude missing or inconsistent data. Data were entered, cleaned and analyzed using IBM SPSS Statistics version 23. Descriptive statistics were computed using frequencies with percentages for categorical variables and median and interquartile range (IQR) for continuous variables. The binary logistic regression model was used to model the association between outcome and independent variables. The final model was assessed for multicollinearity using Variance Inflation Factor (VIF) and goodness of fit using Hosmer and Lemeshow test for independents which make a variable selection decision at each step of the modeling process [36]. Any variable having a significant univariate test with cut-off point p, 0.25 was a candidate for the multivariate analysis [36]. Adjusted odds ratios (AORs) with 95% confidence intervals (CIs) were estimated. A *p*-value of < 0.05 was considered statistically significant. During the analysis phase, each categorical variable having five or more cells were not merged in the categorization of variables.

### Ethical considerations

This research was conducted after obtaining ethical clearance from the Health Research Ethics Review Committee (HRERC) of the College of Health Sciences, Mekelle University. The study was conducted with written and oral consent that assures the willingness of each participant to participate in the study. The pregnant women who were unwilling to participate in the study were respected and only those who were willing to participate in the study were recruited. Each participant signed an informed consent form. Confidentiality and privacy of the pregnant woman were also kept protected.

## Results

### Overview of the status of physical activity

This study has used complete data collected from 299 pregnant women in the analysis phase; 6 incomplete questionnaires were excluded.

Two hundred and ninety-nine women participated in this study, 79.3% (237) were classified as sedentary based on results on the adapted PPAQ. Because 95% of the sample studied attended public antenatal clinics, a comparison across sites, public compared to private clinics was not performed, the relationship of the type of facility to PA status could not be determined. The following provides specific details on the significant associated factors that were related to PA status.

### Socio-demographic characteristics of pregnant women

This study included 299 pregnant women. Nearly half of pregnant women; 148 (49.5%) were between the ages of 16 to 25 years and 289 (96.7%) pregnant women were Tigrian. Only 122 (40.8%) had a high school level of education, 281 (94%) living in the urban area and 265 (88.6%) women reported that their religious affiliation was Orthodox Christian. Of the pregnant women who were surveyed, 262 (87.6%) were married and more than half of the study participants, 168 (56.2%) were women who did not work outside the home. Participants’ socio-demographic characteristics of pregnant women are presented in (Table 1).

**Table 1:** Socio-demographic characteristics of pregnant women attending antenatal care at public and private health facilities in Mekelle, Ethiopia, May 2018 (*n* = 299)

Variables	Frequency, n (%)
Age group (years)	
16–25	148 (49.5)
26–35	136 (45.5)
≥ 36	15 (5.0)
Ethnicity	
Tigray	289 (96.7)
Others^a^	10 (3.3)
Level of education	
Unable to read and write	21 (7.0)
Non-formal education	22 (7.4)
Primary school (1–8 grades)	51 (17.1)
High school (9–12 grades)	122 (40.8)
College/university and above level	83 (27.8)
Residence	
Rural	18 (6.0)
Urban	281 (94.0)
Religion	
Orthodox	265 (88.6)
Muslim	30 (10.0)
Others^b^	4 (1.3)
Marital status	
Married	262 (87.6)
Not married	37 (12.4)
Occupational (employment) status	
Government employee	41 (13.7)
Private institution/business	38 (13.0
women who did not work outside the home	168 (56.2)
Student	11 (3.7)
Merchant	37 (12.4)

Others^a^Amhara, Oromo and Gurage; Others^b^ Adventist, Catholic and Protestant

### Obstetric and health characteristics of pregnant women

Just over half of the women 154 (51.5%) were in their third trimester, women had a BMI of 210 (72.7%) and 131 (43.8%) of the pregnant women were nulliparous. 284 (95.0%) were attending antenatal care at a public health facility most of the pregnant women 233 (77.9%) were spending less than an hour to travel to the health facility, 292 (97.7%) had a history of feeling healthy before getting pregnant and 178 (59.5%) had no current symptoms of discomfort. See other corresponding characteristics in (Table 2).

**Table 2:** Obstetric and health characteristics of pregnant women attending antenatal care at public and private health facilities in Mekelle, Ethiopia, May 2018 (*n* = 299)

Variables	Frequency, n (%)
Stage of pregnancy	
First trimester ≤13 weeks	34 (11.4)
Second trimester [13–24, 26] weeks	111 (37.1)
Third trimester ≥28 weeks	154 (51.5)
Prenatal visits	
Public health facility	284 (95.0)
Private health facility	15 (5.0)
Parity	
0 child	131 (43.8)
1 child	79 (26.4)
2 children	47 (15.7)
> 2 children	42 (14.0)
Travel time to a health facility	
< 60 min	233 (77.9)
An hour or more (≥60 min)	66 (22.1)
History of health status	
Feel healthy	292 (97.7)
Sick/ill	7 (2.3)
Symptoms of discomfort	
Yes	121 (40.5)
No	178 (59.5)
Pre-gravid work status	
Yes	149 (49.8)
No	150 (50.2)
BMI	
Underweight: < 18.5 kg/m^2^	31 (10.7)
Normal: 18.5–24.99 kg/m^2^	210 (72.7)
Overweight: 25–29.99 kg/m^2^	41 (14.2)
Obesity: ≥30 kg/m^2^	7 (2.4)

### Physical activity characteristics of pregnant women

Of the 299 study pregnant women, 211 (70.6%) had received advice about PA from a medical professional with the majority of the advice associated with PA being received from a nurse or midwife 88 (29.5%). Prior to pregnancy, 227 (75.9%) of the pregnant women reported habitual exercise (the usual practice of any PA) whereas, only 110 (48.5%) of the women reported continuing to exercise during pregnancy. The most common reported reason for not exercising during pregnancy was fear of miscarriage 35 (11.7%). The common source of information about PA during pregnancy came from medical/health institutions 145 (48.5%). Walking was the most commonly reported mode of exercise 258 (86.3%). The physical activity characteristics of pregnant women are presented in (Table 3).

**Table 3:** Physical activity characteristics of pregnant women attending antenatal care at public and private health facilities in Mekelle, Ethiopia, May 2018 (*n* = 299)

Variables	Frequency n (%)
Exercise advise from a medical professional	
Yes	211 (70.6)
No	88 (29.4)
Exercise advised by	
Medical doctor	50 (16.7)
Nurse/midwives	88 (29.5)
Health officer	1 (0.3)
Health extension package	72 (24.1)
Method of education on exercise advice	
Individual	181 (60.5)
In a group of other pregnant women	29 (9.8)
Brochure	1 (0.3)
A habit of exercise before pregnancy	
Yes	227 (75.9)
No	72 (24.1)
A habit of exercise while pregnant	
I continued doing exercises	110 (36.8)
I stopped the exercises	45 (15.1)
I continued to exercise but slowed down	72 (24.1)
Reasons stopped exercise activities during pregnancy	
Fear of miscarriage	35 (11.7)
Too Tired	5 (1.7)
Having no time	3 (1.1)
Discomfort	1 (0.3)
Dislikes exercise	1 (0.3)
Comfortable environment	
Yes	231 (77.3)
No	68 (22.7)
Reasons for lack of comfortable environment	
Lack of a safe and secure environment	48 (16)
Cold temperature	5 (1.7)
Lack of accessibility to do exercise	10 (3.3)
Others	5 (1.7)
Self-evaluation of physical activity level prior to pregnancy	
Low	40 (13.4)
Moderate	237 (79.3)
High	22 (7.4)
Self-evaluation of current physical activity	
Less active	123 (41.1)
Increased activity	29 (9.7)
No change	147 (49.2)
Habitual (usual) exercises with husband or partner	
Yes	193 (64.8)
No	106 (35.2)
Sources of information about exercise^a^	
Books and newspapers	13 (4.3)
Friends or relatives	69 (23.1)
Multi-Media	89 (29.8)
Medical/Health institutions	145 (48.5)
Public announcement	6 (2.0)
The person who encouraged you to exercise^a^	
Husband or partner	54 (18.1)
Medical staff	36 (12.0)
Other family members or friends	6 (2.0)
Herself	214 (71.6)
Preferred mode of exercise^a^	
Walking slowly	258 (86.3)
Dancing	17 (5.7)
Prenatal exercise class	14 (4.7)
Swimming	2 (0.7)
Walking quickly	11 (3.7)
Climb hill	2 (0.7)
Running slowly	8 (2.7)
Running quickly	1 (0.3)

^a^ could choose more than one

### Physical activities across trimesters

This study revealed that pregnant women had more or less similar median total energy expenditure across the first, second and third trimesters (3.23 vs 3.35 vs 3.23 MET h/week) respectively. The median energy expenditures on light activities during the three trimesters were 1.61 vs 1.78 vs 1.80 respectively. The amount of energy spent on household activities was also found across first, second and third trimesters (1.33 vs 1.59 vs 1.83 MET h/week) respectively. Physical activities across trimesters are presented in (Table 4).

**Table 4:** Physical activities across trimesters attending antenatal care at public and private health facilities in Mekelle, Ethiopia, May 2018 (*n* = 299)

Physical activities (MET-h/week)	1ST trimester ≤13 weeks (*n* = 34)	2nd trimester (14– 27) weeks (*n* = 111)	3rd Third trimester ≥28 weeks (*n* = 154)
Median (IQR)			
Total energy expenditure per week	3.23 (2.36)	3.35 (2.53)	3.23 (2.17)
By activity intensity(*n* = 299)			
Sedentary	0.62 (0.66)	0.58 (0.88)	0.51 (0.63)
Light	1.61 (1.19)	1.78 (1.06)	1.80 (1.31)
Moderate	0.66 (1.58)	0.77 (1.16)	0.59 (1.18)
Vigorous	0.00 (0.00)	0.00(0.00)	0.00 (0.00)
By type of activity			
Household/caregiving	1.33 (2.02)	1.59 (1.44)	1.83 (1.52)
Occupational (*n* = 130)^a^	1.41 (1.8)	1.48 (1.07)	1.44 (1.03)
Sports/exercise	0.10 (0.15)	0.07 (0.1)	0.09(0.13)
n (%)			
Meet the guideline, n (%)	2 (5.9)	12 (10.8)	11 (7.1)

^a^For occupational activity: 1st trimester = 18, 2nd trimester = 56, and 3rd trimester = 56

### Bivariate and multivariate analysis of factors associated with PA status among pregnant women

The bivariate analysis of the present study revealed that twelve independent variables were a candidate for the final model analysis with cut-off points (*p* < 0.25). These variables are; age group, level of education, marital status, occupational status, stage of pregnancy, parity, travel time to a health facility, symptoms of discomfort, exercises advice from a medical professional, habit of exercise while pregnant, self-evaluation of PA level prior to pregnancy and self-evaluation of current physical activity.

Multivariate analysis revealed that several independent predictors were significantly associated with PA status; Pregnant women in the age group of 26–35 years were 2.69 times more likely to be sedentary (AOR: 2.69, 95% CI: 1.07–6.78) compared with those in the age group of 16–25 years. Pregnant women who had a non-formal education were 13.50 times more likely to be sedentary (AOR: 13.50, 95% CI: 2.65–68.91) compared to those who had graduated from college/university and above level. However, pregnant women who were married were less likely to be sedentary (27%) compared to those who were not married (91%) (AOR: 0.26, 95% CI: 0.09–0.73). Pregnant women who did not work outside the home were 5.23 times more likely to be sedentary (AOR: 5.23, 95% CI: 1.34–20.38) compared to those who were governmentally employed. Pregnant women who had two children were less likely to be sedentary compared to those who had no child regardless of employment status (AOR: 0.13, 95% CI: 0.03–0.59). Pregnant women who traveled an hour or more to arrive at a health facility were less likely to be sedentary compared to those who traveled less than an hour (AOR: 0.31, 95% CI: 0.11–0.89). Women who reported that they exercised regularly during pregnancy but slowed down their overall activity were 2.28 times more likely to be sedentary (AOR: 2.28, 95% CI: 1.03–5.04) compared to those who were continued to exercise while pregnant and maintained their pre-pregnancy activity status. The detailed analysis of factors associated with PA status among pregnant women is presented in (Table 5).

**Table 5:** Bivariate and multivariate analysis of factors associated with physical activity status among pregnant women attending antenatal care at public and private health facilities in Mekelle city, Tigray, Ethiopia, 2018 (*n* = 299)

Variables	Sedentary (237) n (%)	Active (62) n (%)	COR (95% CI)	AOR (95% CI)	*P*-Value
Age group (years)					
16–25	121 (40.5)	27 (9)	(ref)	(ref)	
26–35	106 (35.5)	30 (10)	1.27 (0.71,2.27)	2.69 (1.07, 6.78)	0.036*
> =36	10 (3.3)	5 (1.7)	2.24 (0.71,7.09)	6.66 (0.93,7.98)	0.060
Level of education					
Unable to read and write	17 (5.7)	4 (1.3)	1.27 (0.37,4.38)	2.08 (0.49,8.87)	0.324
Non-formal education	11 (3.7)	11 (3.7)	5.39 (1.93,14.99)	13.5 (2.65,68.91)	0.002**
Primary school (1–8 grades)	41 (13.7)	10 (3.3)	1.31 (0.53,3.26)	1.73 (0.53,5.64)	0.366
High school (9–12 grades)	98 (32.8)	24 (8)	1.32 (0.63,2.77)	2.02 (0.78,5.23)	0.146
College /university and above level	70 (23.4)	13 (4.4)	(ref)	(ref)	
Marital status					
Married	211(70.6)	51 (17)	0.57 (0.27,1.23)	0.26 (0.09,0.73)	0.011**
Not married	26 (8.7)	11 (3.7)	(ref)	(ref)	
Occupational (employment) status					
Government employee	37 (12.4)	4 (1.3)	(ref)	(ref)	
Private business	33 (11)	9 (3.1)	2.39 (0.66,8.69)	3.32 (0.68,16.16)	0.137
women who did not work outside home	124 (41.5)	44 (14.7)	3.30 (1.12,9.79)	5.23 (1.34,20.38)	0.017*
Student	10 (3.4)	1 (0.3)	0.93 (0.09,9.23)	0.95 (0.07,12.27)	0.966
Merchant	33 (11)	4 (1.3)	1.12 (0.26,4.84)	2.06 (0.37,11.53)	0.409
Stage of pregnancy					
First trimester ≤13 weeks	30 (10)	4 (1.3)	(ref)	(ref)	
Second trimester (14–27) weeks	92 (30.8)	19 (6.4)	1.55 (0.49,4.91)	1.52 (0.34,6.74)	0.582
Third trimester ≥28 weeks	115 (38.5)	39 (13.0)	2.54 (0.84,7.68)	2.04 (0.49,8.29)	0.322
Parity					
0 child	102 (34.1)	29 (9.7)	(ref)	(ref)	
1 child	65 (21.8)	14 (4.7)	0.76 (0.37,1.54)	0.69 (0.29,1.69)	0.429
2 children	40 (13.4)	7 (2.3)	0.62 (0.25,1.52)	0.13 (0.03,0.59)	0.008**
> 2 children	30 (10.0)	12 (4.0)	1.41 (0.64,3.09)	0.56 (0.13,2.38)	0.435
Travel time to a health facility					
< 60 min	179 (59.9)	54 (18.0)	(ref)	(ref)	
(≥60 min)	58 (19.4)	8 (2.7)	0.46 (0.21,1.02)	0.31 (0.11,0.89)	0.030*
Symptoms of discomfort					
Yes	103 (34.5)	18 (6.0)	1.88 (1.03,3.44)	1.74 (0.77,3.95)	0.186
No	134 (44.8)	44 (14.7)	(ref)	(ref)	
Exercise advices from a medical professional					
Yes	163 (54.5)	48 (16)	0.64 (0.33,1.24)	0.60 (0.24,1.52)	0.281
No	74 (24.8)	14 (4.7)	(ref)	(ref)	
A habit of exercise during pregnant					
I continued doing exercises	91 (30.4)	19 (6.3)	(ref)	(ref)	
I stopped the exercises	37 (12.4)	7 (2.3)	0.91 (0.35,2.34)	0.97 (0.35,2.70)	0.960
I continued to exercise but slowed down	51 (17.1)	23 (7.7)	2.20 (1.09,4.43)	2.28 (1.03,5.04)	0.041*
Self-evaluation of PA level prior to pregnancy					
Low	36 (12.1)	4 (1.3)	0.50 (0.11,2.23)	0.37 (0.05,2.60)	0.319
Moderate	183 (61.2)	54 (18.1)	1.33 (0.43,4.09)	0.82 (0.19,3.44)	0.785
High	18 (6.0)	4 (1.3)	(ref)	(ref)	
Self-evaluation of PA changes during pregnancy					
Less active	96 (32.1)	27 (9.0)	0.97 (0.55,1.73)	1.05 (0.45,2.46)	0.918
Increased activity	27 (9.0)	2 (0.7)	0.26 (0.06,1.13)	0.37 (0.07,2.04)	0.254
No change	114 (38.2)	33 (11.0)	(ref)	(ref)	

## Discussion

The purpose of this study was to assess PA status and associated factors among pregnant women attending antenatal care at public and private health facilities in Mekelle, Ethiopia. Sedentary PA status was highly prevalent among pregnant women living in Mekelle and the Tigrai region of Ethiopia. Associated factors among pregnant women who had sedentary PA status were married, did not work outside the home and self-reported decreased travel time to the health facility. Associated factors commonly found in pregnant women with active PA status were in the age group (26–35 years old), had 2 children and a non-formal educational level. The findings in our study are consistent with studies conducted in Jimma, Ethiopia (76.4%) [31] and Chili (83%) [22], both studies included participants from similar socioeconomic and educational levels. In contrast, the reported sedentary PA status is higher than findings that have been documented in higher socioeconomic settings, Denmark (6 to 29%) [13], in South Africa 30.8% [30], and 44% [27] and Nigeria 49.0% [25]. Our finding indicates that women in our population that have higher socioeconomic status and education are less active during pregnancy compared with pregnant women living in developed countries. Another possible explanation could be due to an epidemiological and nutritional transition that has occurred in sub-Saharan African countries, including Ethiopia, where lifestyle and behavioral changes, have taken place as a product of both modernization and urbanization that promote physical inactivity, increased adult weight gain, and risk of developing cardiovascular disease [37].

Our study also revealed that the energy expenditure among pregnant women across first, second and third trimesters was significantly low (1.33 vs 1.59 vs 1.83 measured by MET-h.wk-1) respectively; where, most of the energy was expended on household and caregiving activities. This low energy expenditure is comparable to a similar study conducted in Jimma, Ethiopia [31]. However, it was significantly lower than the studies done in Portugal (115.085 vs 97.530 vs 96.509 (MET.h.wk-1)) [20] and Nigeria (75.9 MET-h.week-1) [25]. One possible explanation for this observation is more than half (51.5%) of pregnant women in our cohort were in their third trimester at the time of the data collection period. The third trimester is a period when pregnant women usually go on maternity leave which may make them spend more time at home than at work. Another possible reason is more than half (56.2%) of the pregnant women were women who did not work outside the home thus, possibly spending more time on household activities rather than work activities, exercise or sports activity.

Of the study participants, only 25(8.4%) of the pregnant women met the international recommended guideline for PA (≥150 min moderate-intensity exercise per week) during pregnancy. This finding is significantly lower compared to similar studies conducted in China (11.1%) [17], Ireland (21.5%) [16] and USA (22.9, and 94.5%) [11, 38]. The wide range of differences among those studies could be due to several issues. As an example, Smith et al. in the USA [38] reported that women had a higher level of awareness, economic status, better motivation and fewer perceived barriers to PA. However, our reported PA was higher compared with a similar study conducted with pregnant women in Jimma, Ethiopia (2.8%) [31].

The present study showed that pregnant women in the age group of 26–35 years were 2.69 times more likely to have sedentary PA status compared to those who were in the age group of 16–25 years. This finding is similar to a study conducted by USA National Health and Nutrition Examination Survey in which the young age group, 16–25 years were less likely to be classified as sedentary PA [12]. In a prospective cohort of Britain women, younger women were less likely to decrease their PA levels during pregnancy [14]. A possible reason for this similarity could be because of pregnant women of younger ages in Tigrai, were more actively engaged in different household and occupational activities during pregnancy compared to older women. In Ethiopia, religious belief and culture have an impact on social values. Specifically, it is common to find within a single household that younger household members would be expected to manage any household or occupational activities as a sign of respect for older household members.

This study revealed that pregnant women who had a non-formal education were 13.50 times more likely to have a sedentary PA compared to pregnant women with higher educational levels (graduated College/university and above). This finding is consistent with a similar study conducted in Southeastern Brazil in which the pregnant women who had higher educational levels were more active compared to pregnant women with lower educational levels [18]. However, this study finding is not consistent with a prospective cohort conducted, in Britain, i.e., women with higher educational levels were more likely to decrease their activity levels during pregnancy [14]. In Brazil, this could be related to the possibility that pregnant women with higher educational levels could have more access to knowledge about the benefits of PA whereas, in Britain, those who were highly educated women could have done less household- or occupation-related activities during their pregnancy, and were more likely to spend time on leisure-time PA [14].

This study observed that pregnant women who were married were less likely to be sedentary compared to those who were not married. This finding is consistent with studies conducted in South African, i.e., being married was positively associated with PA [6] and in Britain cohort pregnant women in which women who were married were more active compared to those who were not married [14].

This study also showed that pregnant women who had two children were less likely to be sedentary compared to those who had no child. This study is consistent with studies conducted in southeastern of Brazil in which pregnant women who had two or more children were significantly associated with the increased PA [18] and in Nigeria, i.e., an increasing number of children have increased the probability of being physically active at a moderate level [25]. The relation between an increasing number of children and the possibility of higher PA could be; because pregnant women with more children were a likely higher level of activity required for mothering to be more active compared to those who had no children [25].

This study revealed that pregnant women who traveled an hour or more to arrive at a health facility were less likely to be sedentary compared to those who traveled less than an hour. The possible explanation could be; 86.3% of pregnant women were performing walking as their primary mode of PA. Thus, those who were traveling long distances may have also had chosen walking on foot. Similarly, studies conducted in the USA [11], Britain [23], Portugal [29], Brazil [30], Zambia [14, 18], South Africa [6, 20] showed that walking was the most commonly reported type of PA (29 to 82.2%), during pregnancy. The current study also observed that pregnant women who were continued to walk but slowed down (walked less than the usual) were 2.28 times more likely to experience sedentary PA compared to those who continued exercising while pregnant. This finding could possibly be reflective of the concerns that pregnant women have about the safety of exercise causing anxiety, fear of falling or miscarriage as a deterrent to activity.

### Limitations of this study

There were some limitations to the present study. First, the study’s findings rely on self-reported rather than objective measurements; hence, there may be a bias in the reported results. Second, data from this cross-sectional study did not illustrate exercise conditions (patterns) throughout pregnancy only at one point in time for any one participant. Finally, there was not enough data from a private institution to determine if there is a difference based on the location of public versus private. Future research should include objective methods for assessing activity ie: use of a pedometer or accelerometer as well as, include a process where data collection is done throughout the pregnancy.

## Conclusion

Self-reported sedentary PA status was highly prevalent. Pregnant women in the age group of 26–35 years, attending non-formal education, and women did not work outside the home were positively associated with a higher risk of being sedentary; however, those who married, had two children, and traveled to health facilities in less than an hour were negatively associated with the PA status. It would be better to recommend that pregnancy care providers educate women about the benefits of exercise during pregnancy and that they should continue where possible to be physically active. The partners or family members should be work together in increasing the PA of pregnant women especially experienced women who did not work outside the home with a history of sedentary PA status. More effort is needed among health professionals who interact with pregnant women to remain aware of the importance of exercise promotion.

Policymakers and other stakeholders (Tigrai regional health bureau, Mekelle University, local NGOs working with pregnant women and societies at large) should encourage PA interventions during antenatal care and target family members to improve the health benefits of pregnant women. They should also consider providing PA guidelines combined with education on the benefits of PA for pregnant women. Furthermore, a longitudinal follow-up (interventional) study on the effect of PA during pregnancy with health outcomes for both mothers and offspring is recommended.

## Data Availability

All data which are relevant to this study such as analysis and conclusions were made are available and supplementary data will be offered upon based on a request.

## Abbreviations

ACOG: American College of Obstetricians and Gynecologists
AEE: Activity Energy Expenditure
AOR: Adjusted Odds Ratio
B.Sc.: Bachelor of Science
BMI: Body Mass Index
CHS: College of Health Sciences
CI: Confidence Interval
COR: Crude Odds Ratio
EDHS: Ethiopian Demographic and Health Survey
GDM: Gestational Diabetes Mellitus
KG: Kilo Gram
KJ: Kilo Joule
MET: Metabolic Equivalent Task
MET.h.wk-1: Metabolic Equivalent Task hour per week
MU: Mekele University
NGO: Non-Governmental Organization
PA: Physical Activity
PAL: Physical Activity Level
PPAQ: Pregnant Physical Activity Questionnaire
TRHB: Tigrai Regional Health Bureau
U.S.A: United States of America
